# A Truncated 14-Amino-Acid Myelin Protein-Zero-Targeting Peptide for Fluorescence-Guided Nerve-Preserving Surgery

**DOI:** 10.3390/biom13060942

**Published:** 2023-06-05

**Authors:** Nataliia Berehova, Maarten P. van Meerbeek, Samaneh Azargoshasb, Danny M. van Willigen, Leon J. Slof, Saaedeh Navaei Lavasani, Matthias N. van Oosterom, Fijs W. B. van Leeuwen, Tessa Buckle

**Affiliations:** 1 Interventional Molecular Imaging Laboratory, Department of Radiology, Leiden University Medical Center, 2333 ZA Leiden, The Netherlands; 2 Design and Prototyping, Department of Medical Technology, Leiden University Medical Center, 2333 ZA Leiden, The Netherlands; 3 Department of Head and Neck Surgery, Division of Surgical Oncology, Netherlands Cancer Institute—Antoni van Leeuwenhoek Hospital, 1066 CX Amsterdam, The Netherlands

**Keywords:** fluorescence-guided surgery, nerve imaging, head and neck surgery, receptor targeting, myelin, tracer development

## Abstract

Background: The occurrence of accidental nerve damage during surgery and the increasing application of image guidance during head-and-neck surgery have highlighted the need for molecular targeted nerve-sparing interventions. The implementation of such interventions relies on the availability of nerve-specific tracers. In this paper, we describe the development of a truncated peptide that has an optimized affinity for protein zero (P0), the most abundant protein in myelin. Methods and Materials: Further C- and N-terminal truncation was performed on the lead peptide Cy5-P0_101–125_. The resulting nine Cy5-labelled peptides were characterized based on their photophysical properties, P0 affinity, and in vitro staining. These characterizations were combined with evaluation of the crystal structure of P0, which resulted in the selection of the optimized tracer Cy5-P0_112–125_. A near-infrared Cy7-functionalized derivative (Cy7-P0_112–125_) was used to perform an initial evaluation of fluorescence-guided surgery in a porcine model. Results: Methodological truncation of the 26-amino-acid lead compound Cy5-P0_101–125_ resulted in a size reduction of 53.8% for the optimized peptide Cy5-P0_112–125_. The peptide design and the 1.5-fold affinity gain obtained after truncation could be linked to interactions observed in the crystal structure of the extracellular portion of P0. The near-infrared analogue Cy7-P0_112–125_ supported nerve illumination during fluorescence-guided surgery in the head-and-neck region in a porcine model. Conclusions: Methodological truncation yielded a second-generation P0-specific peptide. Initial surgical evaluation suggests that the peptide can support molecular targeted nerve imaging.

## 1. Introduction

Image-guided applications play an important role in the current progression realized in the field of head-and-neck surgery [1,2,3,4]. A prime example of such an approach is found in the surgical resection of sentinel nodes, a procedure that serves as a secondary means for the identification of (micro)metastatic tumor spread [1,2,5,6]. An upcoming application is found in the visualization of primary tumor margins [7,8,9,10,11]. Unfortunately, the location of both possibly involved lymph nodes and the primary tumor can coincide with the location of nerves within the complex anatomy of the head and neck area (e.g., the hypoglossal, lingual, and vagus nerves and the marginal mandibular trunk of the facial nerve; [12,13]). As a result, the occurrence of accidental surgically induced nerve damage is not uncommon. For instance, injury to the laryngeal nerve or mandibular nerve is seen in 14% of patients undergoing thyroid surgery or neck dissection, respectively [14,15]. Permanent paralysis is even seen in 4–7% of these patients [15]. This underlines that the intraoperative identification of nerves has the potential to serve as an important goal and target for image guidance strategies.

With its superior spatial resolution, multispectral/multiwavelength properties, and real-time imaging capabilities, fluorescence imaging has the potential to support high-level discrimination between target and non-target tissue [16,17]. To achieve fluorescent ‘illumination’ of nerves, nerve-targeting fluorescent tracers are required. Over the last years, a number of such tracers have been developed [18], including non-conjugated fluorescent dyes [19,20,21,22], peptides with either unknown nerve-related or intracellular targets such as myelin basic protein (MBP; [23,24,25]), or peptides that target myelin protein zero (MPZ, P0). The latter of these is the most abundant protein present in myelin [26,27,28]. The pursuit of the targeting of nerve-specific proteins is in line with conventional tumor receptor-targeted molecular imaging strategies [29]. Initial work on the targeting of P0 focused on truncation of the extracellular portion of the homotypic P0 protein into amino acid sequences [30]. This yielded the P0-specific lead compound Cy5-P0_101–125,_ consisting of 26 amino acids that exhibited a binding affinity of 104 ± 17 nM [27]. Dimerization of this peptide (Cy5-(P0_101–125_)_2_; [26]) yielded a 1.5-fold increase in affinity but almost doubled the molecular weight. In vitro and in vivo evaluation using fluorescence immunohistochemistry of stained nerve tissue, blocking and FLIZA studies, and in vivo and ex vivo imaging after local intravenous and intraneural administration showed the robustness of this P0-taregting concept [26].

**Figure 1:**
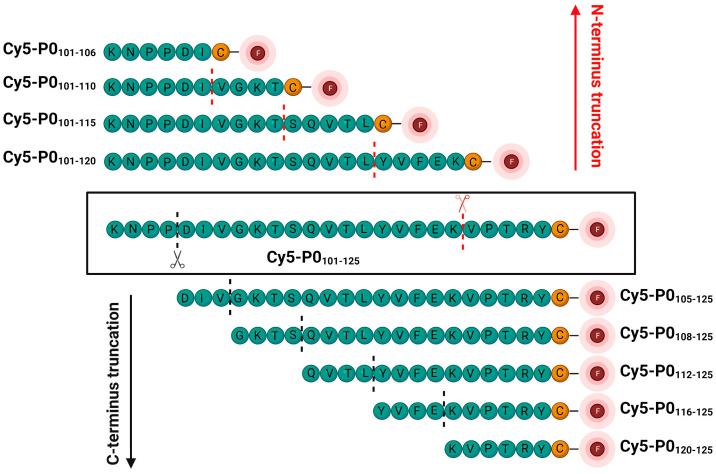
Methodological N- and C-terminal truncation of the lead compound Cy5-P0_101–125_ (center), resulting in N- (red arrow; top) and C-terminal (black arrow; bottom) truncation matrices. Peptide labelling with F (fluorophore) was achieved using a maleimide–cysteine coupling at the N-terminal face of each peptide.

In this paper, we describe an alternative optimization route for the lead compound Cy5-P0_101–125_, namely, through methodological peptide truncation. To achieve this, the lead peptide was alternately truncated from both the C- and N-terminal sides, while systematically functionalizing the peptide with a Cy5-fluorescent label through a maleimide–cysteine coupling at the N-terminal face of the peptide (Figure 1). Following synthesis, the photophysical properties of these compounds were characterized, and their P0 affinity/specificity was determined via flow cytometry and confocal microscopy. Pearson correlation analysis of the peptide characteristics and binding interactions observed in the crystal structure of P0 was used to unravel the structure–activity relationship. The peptide sequence that showed the most favorable characteristics was subsequently labelled with a near-infrared Cy7 dye and applied during head-and-neck nerve surgery in a porcine model using a clinical-grade fluorescence camera.

## 2. Materials and Methods

All chemicals were received from Actu-All Chemical (Oss, The Netherlands), Sigma Aldrich (St. Louis, MO, USA), Tokyo Chemical Industry (Tokyo, Japan), Biosolve BV (Valkenswaard, The Netherlands), and VWR Chemicals (Solon, OH, USA) and used without further purification. DMF and DMSO were dried over 4 Å molecular sieves for at least 24 h prior to use. Preparative high-pressure liquid chromatography (prep-HPLC) was performed on a Waters HPLC system (Waters Chromatography B.V., Etten-Leur, The Netherlands) using a 2545 quaternary gradient module pump and a 2489 UV detector. A Dr. Maisch ReproSil-Pur 120 C18-AQ 10 μM (250 mm × 20 mm) column (Dr. Maisch HPLC GmbH, Ammerbuch-Entringen, Germany) was applied with an operating flow rate of 12 mL/min. Analytical HPLC was performed on a Waters Acquity UPLC-MS system using a Acquity UPLC photodiode array detector, an SQ Detector mass spectrometer, and a flow rate of 0.5 mL/min (Waters BEH C18 130 Å 1.7 mm (100 mm × 2.1 mm) column). Lyophilization was performed using a VaCo 2-II lyophilizer (Zirbus technology GmbH, Bad Grund (Harz), Germany). Absorption spectrometry was performed using a UV1280 UV–vis spectrometer (Shimadzu, Kyoto, Japan), and fluorescence spectrometry was performed using an LS55 (Perkin Elmer, Waltham, MA, USA). Fluorescence confocal imaging was performed using an SP8 WLL confocal microscope (Leica Microsystems, Wetzlar, Germany). The obtained images were analyzed using Leica Confocal Software (Leica Microsystems). The mean fluorescence intensity values per sample were measured using an LSRII flow cytometer (BD Biosciences, Franklin Lakes, NJ, USA) with APC-A settings (635 nm laser and 750 nm long-pass filter) for a Cy5 dye. Image acquisition and processing for the assessment of P0 staining were performed using LASX software (Leica Application Software Suite 4.8). Image analysis (3D) was performed using Image J [26]. For the affinity assessment, fluorescence was measured using a FACSCanto II flow cytometry device (BD Biosciences, Franklin Lakes, NJ, USA) in the APC-A channel, and the accompanying FlowJo^TM^ v5 software (BD Biosciences, Franklin Lakes, NJ, USA) was used for further analysis. Figure 1 was created using BioRender (https://biorender.com/, accessed on 30 May 2023). Feature clustering and Pearson correlation assessment were performed using MATLAB 9.9.0.1495850 (R2020b) software (The MathWorks Inc.: Natick, MA, USA). For crystal structure analysis, RCSB PDB (https://www.rcsb.org/) was used.

### 2.1. Synthetic Procedures

#### 2.1.1. Dye Synthesis

The sequences of P0 peptides, Sulfonate-(SO_3_)Cy5(SO_3_)-Maleimide, and its precursors were synthesized according to earlier reported procedures [26,31,32]. For synthesis of the Cy7 analogue, the precursor Sulfonate-(SO_3_)Cy7(SO_3_)-COOH [33] was dissolved in dry DMSO (5 mL), followed by the addition of PyBOP (2.5 eq), N-(2-aminoethyl)maleimide (1.2 eq), and N-methyl morpholine (4 eq). The reaction mixture was stirred for 3 h at r.t. The crude solution was purified by preparative HPLC employing a gradient of 25 → 95% acetonitrile. Yield 65.8%. m/z: Calcd for C_43_H_50_N_4_O_12_S_3_^2-^ 911, 07, found 913.5.

#### 2.1.2. General Synthetic Procedure for the Fluorescent C-Terminal Labelling of Truncation Peptides

A stock solution of Maleimide-(SO_3_)Cy5(SO_3_)-Sulfonate (12, 4 mM) in Mili-Q (1) was made. An excess of truncated P0 peptide (Figure 1; 1.4 eq) was dissolved in phosphate-buffered saline (PBS) (pH 7.4, 100 mM), followed by the addition of 1 eq of the dye from the stock solution. The mixture was sonicated and allowed to react at r.t. for 4 h. The crude product was purified via preparative HPLC (gradient 15 → 95% acetonitrile in Mili-Q over 30 min). A blue, fluffy compound was obtained after lyophilization for all truncated Cy5-labeled tracer derivatives.

For creation of the Cy7 derivative, peptide (1 eq) was dissolved in degassed buffer HEPES at pH 7.4. Sulfonate-(SO_3_)Cy7(SO_3_)-Maleimide (20 eq) was dissolved in DMSO. A 100× molar excess of TCEP was added to the solution with the peptide, followed by the addition of maleimide. The reaction mixture was flushed with nitrogen, mixed thoroughly, and kept at room temperature overnight. The crude product was purified via preparative HPLC, employing a gradient of 5 → 95% acetonitrile in Mili-Q. A green, fluffy compound (3a) was obtained after lyophilization. Cy5-P0_101–125_: 6.4 mg (yield 36.3%), m/z [M+3H—979.7]; Cy5-P0_101–120_: 4.4 mg (yield 24%), m/z [M+3H − 1999.3]; Cy5-P0_101–115_: 5.1 mg (yield 34.5%), m/z [M+3H − 877.4]; Cy5-P0_101-110_: 1.4 mg (yield 15%), m/z [M+2H − 1051.0]; Cy5-P0_101–106_: 0.87 mg (yield 42%), m/z [M+2H—858.15]; Cy5-P0_105–125_: 0.8 mg (10.3%), m/z [M+3H+ Na+—1165.3]; Cy5-P0_108-125_: 1.7 mg (yield 16.2%), m/z [M+3H − 1050.7]; Cy5-P0_112–125_: 0.4 mg (yield 5%), m/z [M+3H − 925.6]; Cy5-P0_116–125_: 1.7 mg (yield 15.8%), m/z [M+2H − 1167.6]; Cy5-P0_120–125_: 2.8 mg (yield 20.9%), m/z [M+2H − 898.0]; Cy7-P0_112–125_: 0.5 mg (yield 16.5%), m/z [M+2H − 1400.8]. See Figures S1–S12 for mass spectra of the synthesized compounds.

### 2.2. Experimental Procedures

#### 2.2.1. Assessment of P0 Staining via Fluorescence Confocal Microscopy

The P0 specificity of Cy5-P0_101–125_ was previously determined in P0-expressing myelinating Schwannoma cells (RT4 D6P2T myelinating Schwannoma cells (ATCC^®^ CRL-2768™; [34])) and non-P0-expressing cells, using P0 antibodies and a fluorescent derivative of the extracellular portion of P0 [27]. P0-expressing RT4 D6P2T cells were grown in Dulbecco’s modified Eagle medium (Life Technologies, Paisley, UK) containing penicillin, streptomycin, and fetal calf serum (All BD Biosciences) at 37 °C and 5% CO2. Cells were trypsinized and seeded onto 35 mm culture dishes that contained a glass insert (MatTek co) one or two days prior to the imaging experiment. One hour prior to imaging, 1 μM Cy5-P_101–125_, Cy5-P0_101–120_, Cy5-P0_101–115_, Cy5-P0_101–110_, Cy5-P0_101–106_, Cy5-P0_105–125_, Cy5-P0_108–125_, Cy5-P0_112–125_, Cy5-P0_116–125,_ Cy5-P0_120–125_, or the original lead compound Cy5-P0_101–125_ [27] was added (incubation at 4 °C; N = 3 per tracer). Imaging was performed as previously described [35]. Image acquisition and processing were performed using LASX software (Leica Application Software Suite 4.8). Image analysis, including (3D) surface plot and co-localization analysis, was performed using Image J [26].

#### 2.2.2. Affinity Assessment via Flow Cytometry

Analysis of the binding affinity (K_D_) of Cy5-P_101–125_, Cy5-P0_101–120_, Cy5-P0_101–115_, Cy5-P0_101–110_, Cy5-P0_101–106_, Cy5-P0_105–125_, Cy5-P0_108–125_, Cy5-P0_112–125_, Cy5-P0_116–125_, and Cy5-P0_120–125_ for P0 was performed using P0-expressing RT4 D6P2T cells and a previously described flow cytometric method [27,35]. Saturation binding experiments were performed for each of the fluorescent peptides in a concentration range of 0–2000 nM. All measurements were performed in triplicate, and experiments were repeated at least two times per tracer. The fluorescence was measured using a FACSCanto II flow cytometry device (BD Biosciences) in the APC-A channel, and the accompanying FlowJo software was used for further analysis. K_D_ assessment was performed using the normalized geometric means, which were fitted with equations in GraphPad Prism 5 software.

#### 2.2.3. Chemical and Photophysical Properties

Determination of the chemical and photophysical properties (LogP, solubility, serum binding, net charge, and brightness) was performed as previously described [27,31,36].

#### 2.2.4. Correlation Assessment and P0-Related Interactions

Cluster analysis was applied based on standardized values for each feature using the clustergram function in MATLAB [37]. An evaluation of P0-related interactions was performed using peptide characteristic and inter- and intra-molecular interactions that were extracted from the crystal structure of the extracellular portion of P0 [38].

#### 2.2.5. In Vivo Imaging of the Optimized Peptide

In vivo experiments in pigs were approved by the ethical board of the University of Ghent (EC2019/79). Pigs were housed at the animal facility at ORSI Academy (Melle, Belgium) until use for nerve imaging experiments during surgical training (weight per animal: approximately 40 kg). Experiments were performed in accordance with the Experiments on Animals Act (Wod, 2014) and the applicable legislation in Belgium, and in accordance with the European guidelines (EU directive no. 2010/63/EU) regarding the protection of animals used for scientific purposes. The pigs were bred and kept in accordance with Belgium law in and by a licensed establishment for the use of experimental animals.

Cy7-P0_112–125_ (0.25 mg, 300 µmol) was evaluated in pigs undergoing open surgery in the head and neck area using a clinical-grade handheld fluorescence camera (N = 2, PDE-mod, Hamamatsu Photonics; [39]). Animals received either intravenous injection (in the jugular vein under ultrasound guidance) or injection directly into the vagus nerve (intraneural administration). In both cases, fluorescence imaging of the vagus nerve and surrounding tissues was performed at 1 h after tracer administration. The animals were maintained under isoflurane anesthesia for the complete duration of the surgical training and subsequent nerve imaging experiments and were euthanized before awakening from the anesthesia. After resection, ex vivo imaging was applied using the same camera system. Image processing was performed using in-house custom-developed software [27,40].

### 2.3. Statistical Evaluation

A statistical evaluation to compare the affinity (K_D_) was performed using unpaired Student’s t-test. Values of *p* < 0.05 were considered significant. Pearson correlation analysis was applied based on standardized feature values according to previously described methods [37].

## 3. Results

### 3.1. Synthesis and Compound Properties

Systematic truncation of the original lead peptide P0_101–125_ resulted in the creation of Cy5-P0_101–120_, Cy5-P0_101–115_, Cy5-P0_101–110,_ Cy5-P0_101–106_, Cy5-P0_105–125_, Cy5-P0_108–125_, Cy5-P0_112–125_, Cy5-P0_116–125_, and Cy5-P0_120−125_ (Figure 1). Assessment of the photophysical properties of these tracer derivatives (Table 1) revealed substantial differences in their LogP values (range: 0.60–1.59). With the exception of Cy5-P0_116–125_ and Cy5-P0_120–125_, truncation resulted in a decrease in serum binding (from 89% to 47–69%). C-terminal truncation of the PTRY_122–125_ amino acid sequence (Cy5-P0_101–120_) resulted in a twofold increase in brightness, a feature that was maintained throughout the C-terminal matrix. A less pronounced effect was seen within the N-terminal truncation matrix, as a similar increase in brightness was only seen for Cy5-P0_105–125_ and Cy5-P0_112–125_. Net charge remained in the range of −1 to −3 for all tracer derivatives.

### 3.2. P0 Staining by Fluorescence Confocal Microscopy

**Figure 2:**
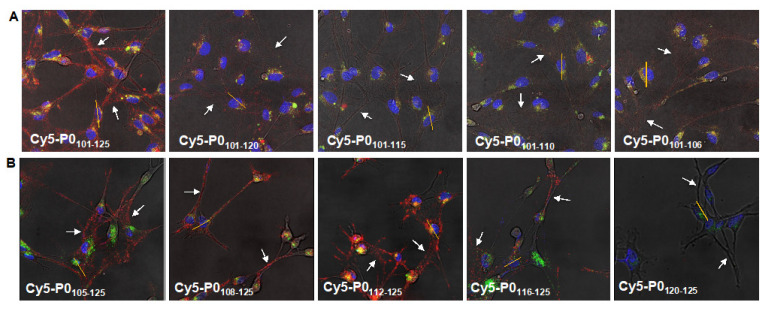
P0-related cellular staining. Two-dimensional fluorescence confocal imaging for assessment of P0-related staining of (**A**) the tracers in the C-terminal matrix (Cy5-P0_101–125_, Cy5-P0_101–120_, Cy5-P0_101–115_, Cy5-P0_101–110_, and Cy5-P0_101–106_) and (**B**) the tracers in the N-terminal matrix (Cy5-P0_105–125_, Cy5-P0_108–125_, Cy5-P0_112–125_, Cy5-P0_116–125_, and Cy5-P0_120–125_) in P0-expressing RT4 Schwannoma cultures. P0-related staining (Cy5) in red, lysosomes in green, and nuclear staining in blue. Yellow line: orientation 3D analysis; white arrows: examples of locations of cellular outgrowth within the sample.

In line with previous reports, the staining of Cy5-P0_101–125_ in P0-expressing RT4 Schwannoma cells [27] was in agreement with the location of P0 on the membrane (P0-related staining in red; Figure 2) and, more specifically, on the cellular outgrowths (white arrows) of the Schwannoma cells.

#### 3.2.1. C-Terminal Matrix

A comparison between the compounds within the C-terminal matrix and the lead compound Cy5-P0_101–125_ revealed differences in localization, as well as P0-related staining intensity (Figure 2A, in red). The level of staining of the cellular outgrowths was shown to decrease with each truncation step, suggesting that C-terminal truncation resulted in a loss of P0 specificity. The differences in the localization of staining were underlined by surface plot analysis of the fluorescence (Figure S13A). The loss of staining of the outgrowths was shown to correspond to an increase in the level of co-localization with the intracellular control staining/non-specific staining (Table S1).

#### 3.2.2. N-Terminal Matrix

In contrast to that with the C-terminal truncation of P0_101–125_, the P0-related staining pattern was maintained during the first three N-terminal truncation steps (Figure 2B). Interestingly, truncation from Cy5-P0_105–125_ to Cy5-P0_108–125_ and Cy5-P0_112–125_ resulted in an increase in P0-related staining intensity. This was corroborated by an increase in peak height and intensity in the corresponding surface plots (Figure S13B). Further truncation (Cy5-P0_116–125_) resulted in a decrease in staining intensity, while no specific staining was observed for Cy5-P0_120–125_. Co-localization with the intracellular control staining was shown to be low for Cy5-P0_105–125_, Cy5-P0_108–125_, and Cy5-P0_112–125,_ while the loss in P0-related staining for Cy5-P0_116–125_ and Cy5-P0_120–125_ again resulted in an increase in the level of co-localization (Table S1).

### 3.3. P0 Affinity Determined by Flow Cytometry

**Figure 3:**
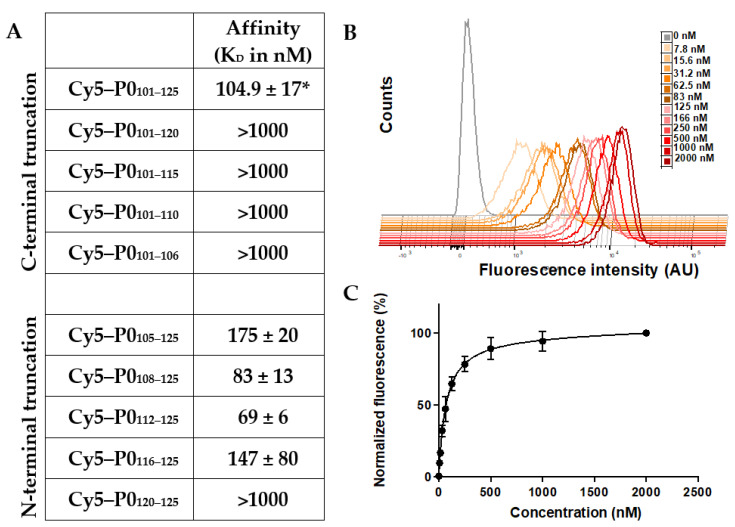
Affinity of truncated peptide matrixes. (**A**) Affinity (K_D_) values for N- and C-terminal truncated peptides determined via saturation binding using P0-expressing RT4 Schwannoma cells. * Obtained from [27]. (**B**) A 3D representation of the increase in signal intensity and (**C**) saturation binding curve over the applied concentration range (0–2000 nM) for the sequence with the highest K_D_, namely, Cy5-P0_112–125_.

Peptides within the C-terminal truncation matrix did not show nanomolar binding affinity for P0 (K_D_; Figure 3A), which is in line with the lack of specific staining seen in Schwannoma cells (Figure 2A). N-truncation did, however, show a significant increase in P0-related affinity from 175 to 83 (*p* = 0.04) and 69 nM (*p* = 0.0001) for Cy5-P0_105–125_, Cy5-P0_108–125_, and Cy5-P0_112–125_, respectively. As an example, a representation of the increase in signal intensity and the resulting saturation binding curve for Cy5-P0_112–125_ are provided in Figure 3C,D. Again, the affinity could be related to P0-related staining (Figure 2B).

Interestingly, solubility (Table 1) seems to be linked to affinity. Tracers with a low affinity of >1000 nM were soluble, while higher affinity tracers (e.g., Cy5-P0_108–125_ and Cy5P0_112–125_) showed relatively low solubility (Table 1). This unexpected effect might be explained through the intrinsic homotypic interactions between P0 molecules, wherein the levels of inter- and intra-molecular interactions can both affect affinity and solubility.

### 3.4. Relationships between Chemical and Biological Features

**Figure 4:**
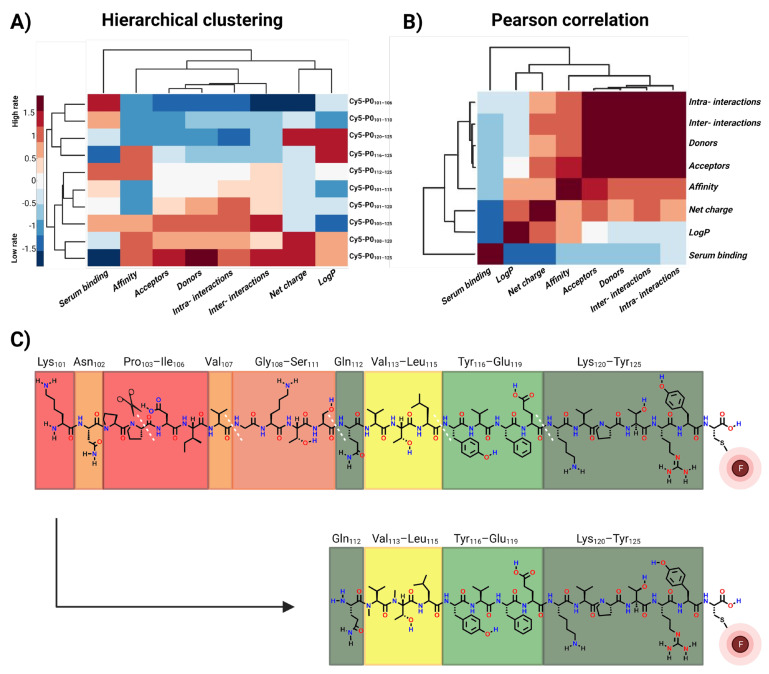
Correlation of P0-related interactions and binding. (**A**) Hierarchical clustering of standardized data values of features of truncated compounds (red—higher rate of the relation between the features, blue—negative relation). (**B**) Pearson correlation. The color bar represents the correlation strength between the tracers’ features (red—positive correlation, blue—negative correlation). (**C**) Lead and truncated P0-targeting peptides Cy5-P0_101–125_ and Cy5-P0_112–125_. Hydrogen donors (blue) and acceptors (red) that support inter- or intra-molecular interactions have been indicated on peptide sequences. The importance of the peptide sequences is color coded, with the most important binders in green and the least important ones in red. F, fluorophore.

Analysis of the crystal structure of the extracellular portion of P0 [38] allowed an investigation of the inter- and intra-molecular interactions between the parental amino acids in this protein (Table 2). Pearson correlation assessment based on the peptide characteristics and the interactions observed in the crystal structure revealed three different subclasses of compounds (Figure 4, *y*-axis). In this classification, an intermediate or low number of acceptor and donor molecules and a low number of supramolecular/intermolecular interactions were found to be the main denominators. These structural features showed a direct correlation with the level of affinity (high number of donors/acceptors = high affinity, low number of donors/acceptors = low affinity). This correlation was also seen for the level of solubility (Table 1).

**Figure 5:**
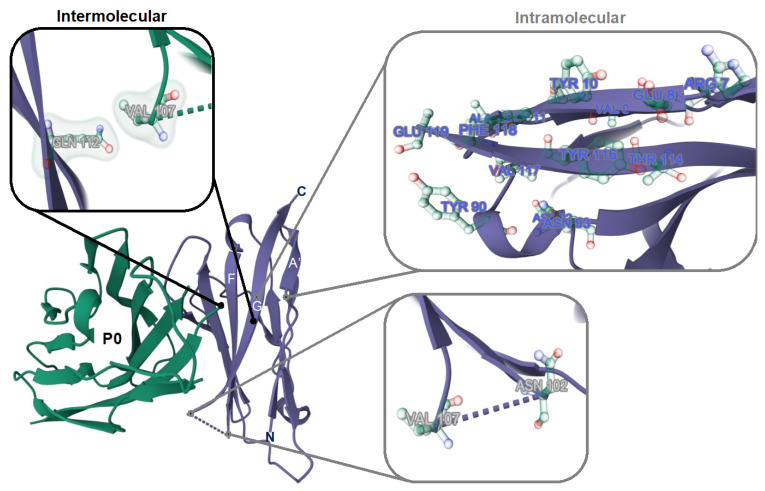
Inter- and intra-molecular amino acid interactions in the G B-helix of P0. Representation of the crystal structure of the extracellular portion of P0 and binding between two P0 molecules (in green and purple). C denotes the C-terminus and N denotes the N-terminus of the P0 molecule. A’, G, and F highlight the involved β sheets. Intermolecular interactions between Q_112_ and V_107_ (top left), intramolecular interaction of TLYVFE_114–119_ with β sheet A’ (top right), and intramolecular interactions between N_102_ and V_107_ are highlighted.

Truncation of the original lead compound Cy5-P0_101–125_, a peptide that largely overlaps with the G B-helix in the extracellular portion of the P0 structure (Figure 5), decreased the number of interactions. This yielded varying effects on the binding affinity of the tracers (Figure 3 and Figure 4). C-terminal truncation of the PTRY_122–125_ amino acid sequence resulted in a loss of affinity. This implies that this sequence has a critical function within the peptide. N-terminal truncation of the KNPP_101–104_ amino acid sequence yielded a 1.67-fold drop in affinity (*p* > 0.0003), suggesting that this sequence plays a role in P0 binding, but one that is less critical.

The crystal structure of P0 indicates that N_102_ can bind to V_107,_ thereby creating a loop within the peptide (Figure 5). Interestingly, truncation of the DIV_105–107_ amino acid sequence again yielded a 2.1-fold rise in affinity (*p* = 0.0002, Figure 3A). This is especially interesting when one realizes that the P0 crystal structure indicates that the amino acids V_107_ and Q_112_ facilitate intermolecular van der Waals interactions (Figure 5; [41]). Further truncation of GKTS1_08–111_ only yielded a modest 1.2-fold (*p* = 0.09) rise in affinity. Removal of the QVTL_112–115_ sequence reduced the affinity by nearly 2.13-fold (*p* = 0.09) and therefore seems to be rather critical for maintaining affinity for P0. Further truncation of YVFE_116–119_, a sequence containing a plurality of H-donors and acceptors, again resulted in a complete loss of affinity. Further analysis of the crystal structure suggests that sequence TLYVFE_114–119_ provides intramolecular binding for the A’ and F B-sheets (Figure 5 (top right); [41]).

### 3.5. In Vivo Imaging of Nerves in the Head and Neck Region

To provide a proof-of-principle of intraoperative real-time near-infrared nerve visualization in an open surgery setting, Cy7-P0_112–125_ was synthesized and evaluated in a porcine model.

**Figure 6:**
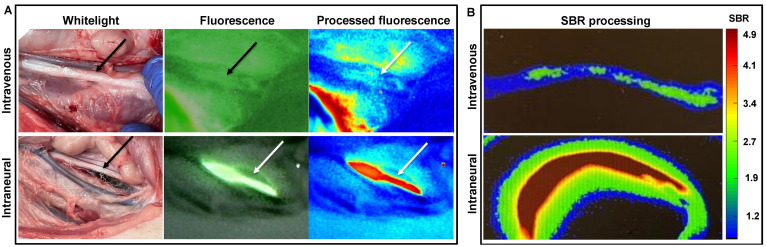
In vivo and ex vivo imaging using Cy7-P0_112–125_. (**A**) In vivo whitelight and fluorescence imaging using a clinical-grade hand-held fluorescence camera after local intravenous or intraneural tracer administration. Arrow: location of the vagus nerve. (**B**) Ex vivo fluorescence-based color-coded intensity-based image-processing evaluation of excised nerve tissue showing fluorescence in green and that allowed evaluation of the signal-to-background ratio (SBR).

Intravenous administration of Cy7-P0_112–125_ in the jugular vein yielded a fluorescence signal in the vagus nerve (Figure 6A, black arrow). However, the signal-to-background ratio was low. Intraneural administration substantially improved the staining (Figure 6A). Ex vivo assessment of excised nerve tissue followed by fluorescence-intensity-based image processing (Figure 6B) confirmed tracer uptake in the nerve after intravenous administration (SBR: 1.9) and after intraneural administration (SBR: 4.9).

## 4. Discussion

Methodological peptide truncation was shown to provide a successful optimization route, resulting in the selection of Cy5-P0_112–125_. This P0-specific peptide showed a 1.5-fold increase in affinity compared to the lead compound Cy5-P0_101–125_ (Figure 3; *p* = 0.01), while a 47% reduction in the number of incorporated amino acids was realized (from 26 (101–125; KNPPDIVGKTSQVTLYVFEKVPTRY) to 14 (112–125; QVTLYVFEKVPTRY), Figure 1). This route not only allowed the generation of a new lead, but also provided increased mechanistic insight into the molecular requirements of P0 binding.

Truncation combined with affinity studies (Figure 3), chemical analysis (Table 1), and P0 crystal structure observations (Figure 3 and Figure 4, and Table 2) gave interesting insights into the (homotypic) peptide binding of the P0 proteins. P0-related affinity seems to be dominated by two primary binding interactions. The first is the apparent anchoring function of the C-terminal VPTRY_121–125_ sequence [41], a feature that was shown to be critical in the positioning of the P0 proteins, and, in particular, the GKTSQVTLYVFE_108–119_ pharmacophore, for binding. (Figure 3). The GKTSQVTL_108–115_ portion of the original lead sequence was shown to have the most prominent effect on P0-related binding. The presence of Q_112_ in this sequence seemingly facilitates the targeting of V_107_, while the remaining amino acids provide a supporting function. More specifically, YVFE_116–119_ on its own did not show affinity for P0, but one can argue that its main role is to help facilitate the positioning of Q_112_.

Previous work on P0-derived myelin-targeting peptides indicates that this target is specific for the peripheral nervous system [27], reducing the risk of late-term toxic effects due to accumulation in the CNS. This feature is a common side effect for small-molecule nerve agents derived from, e.g., the neural imaging agent Pittsburgh compound B [20,22,42,43]. Various studies have put forward local tracer administration as a means to decrease uptake beyond the surgical field [19,27,44]. This strategy is generally employed to help boost the effective local concentration and, as such, facilitate a positive effect on local targeting. In line with our previous work on Cy5-P0_101–125_ [27], the porcine studies in Figure 6 indicate that intravenous injection of Cy7-P0_112–125_ in the vessel leading to the organ of choice did indeed induce staining of the vagus nerve. As previously shown for other targeted tracers, optimization of the dosing and timing is expected to provide higher SBR values, further adding to the utility of this administration strategy [29]. While intraneural administration yielded explicit nerve staining with a high SBR, longitudinal studies are needed to determine whether this administration route could have negative effects on nerve viability.

Surgical visualization of nerves may not only serve head-and-neck surgery, but also help promote nerve sparing in, e.g., neuro-, orthopedic, colorectal, bladder, and prostate surgery. As surgery is moving increasingly towards minimally invasive (robotic) approaches, it is critical that the nerve-specific tracers are compatible with clinical-grade endoscopic camera systems. To this end, the far-red Cy5 dye (l_ex_ = 650 nm, l_em_ = 667 nm) has demonstrated compatibility with a KARL STORZ prototype [40,45], while the near-infrared Cy7 dye (l_ex_ = 750 nm, l_em_ = 777 nm; [16]) is compatible with the Image 1 S Rubina (KARL STORZ) and the Firefly endoscope+ (Intuitive) set-up. These features will promote further dissemination.

## 5. Conclusions

Methodological truncation of the lead compound Cy5-P0_101–125_, combined with assessment of the crystal structure of the extracellular portion of P0 and inter- and intra-molecular binding interactions, resulted in the selection of the optimized tracer Cy5-P0_112–125_. This high-affinity tracer allowed effective P0-related staining in vitro, and its Cy7 derivative was shown to be compatible with clinical-grade fluorescence imaging devices during nerve imaging in a porcine model.

## 6. Patents

European patent application No.16180535.3.

## Figures and Tables

**Table 1:** Chemical and photophysical properties of P0-targeting fluorescent tracers.

	**Solubility** **(** **µ** **M)**	**LogP**	**Serum** **Binding**	**Brightness** **(in HSA)**	**Net Charge**
Cy5-P0_101–125_	172	−1.39 + 0.09	89 + 2%	67,760	−1
Cy5-P0_101–120_	15	−0.99 ± 0.1	66 ± 12%	137,940	−2
Cy5-P0_101–115_	>400	−0.80 ± 0.16	60 ± 9%	217,800	−2
Cy5-P0_101–110_	>400	−0.73 ± 0.12	55 ± 8%	111,320	−2
Cy5-P0_101–106_	>400	−0.95 ± 0.05	47 ± 7%	118,580	−3
Cy5-P0_105–125_	9	−0.60 ± 0.04	56 ± 8%	157,300	−2
Cy5-P0_108–125_	25	−1.34 + 0.03	69 ± 7%	58,080	−1
Cy5-P0_112–125_	71	−1.08 + 0.03	50 + 3%	121,000	−2
Cy5-P0_116–125_	150	−1.58 + 0.03	81 + 5%	60,500	−2
Cy5-P0_120–125_	150	−1.59 + 0.01	71 ± 11%	70,180	−1

HSA = human serum albumin.

**Table 2:** Inter- and intra-molecular interactions.

	No. of Acceptors	No. of Donors	No. of Intramolecular Interactions	No. of Intermolecular Interactions
Cy5-P0_101–125_	40	39	26	3
Cy5-P0_101–120_	29	29	26	2
Cy5-P0_101–115_	24	22	18	2
Cy5-P0_101–110_	16	14	8	1
Cy5-P0_101–106_	10	9	2	0
Cy5-P0_105–125_	36	33	24	3
Cy5-P0_108–125_	32	28	23	2
Cy5-P0_112–125_	26	21	16	2
Cy5-P0_116–125_	20	15	8	1
Cy5-P0_120–125_	13	10	0	1

## Data Availability

The data presented in this study are available on request from the corresponding author. The data are not publicly available due to patent restrictions.

## Supplementary Materials

The following supporting information can be downloaded at: https://www.mdpi.com/article/10.3390/biom13060942/s1, Figure S1: Mass spectrum of Cy5-P0_101–125_. m/z [M+3H—979.7]; Figure S2: Mass spectrum of Cy5-P0_101–120_. m/z [M+3H—1999.3]; Figure S3: Mass spectrum of Cy5-P0_101–115_. m/z [M+3H—877.4]; Figure S4: Mass spectrum of Cy5-C-P0_101–110_. m/z [M+2H –1051.0 ]; Figure S5: Mass spectrum of Cy5-P0_101–106_. m/z [M+2H—858.5]; Figure S6. Mass spectrum of Cy5-P0_105–125_. m/z [M+3H+ Na^+^—1165.3]; Figure S7: Mass spectrum of Cy5-P0_108–125_. m/z [M+2H– 1050.7]; Figure S8: Mass spectrum of Cy5-P0_112–125_.m/z [M+2H—1389.0]; Figure S9: Mass spectrum of Cy5-P0_116–125_. m/z [M+2H—1167.6]; Figure S10: Mass spectrum of Cy5-P0_120–125_. m/z [M+2H—898.0]; Figure S11: Mass spectrum of *Maleimide-(SO_3_)Cy5(SO_3_)Sulfonate*. m/z [M—913.4]; Figure S12: Mass spectrum of Cy7-P0_112–125_. m/z [M+2H—1400.8]; Figure S13: Surface plot analysis fluorescence confocal images; Table S1: Semi-quatitative assessment of co-localization staining P0 tracers and lysosomal and nuclear control staining.
