# Supplementary material for: A Truncated 14-Amino-Acid Myelin Protein-Zero-Targeting Peptide for Fluorescence-Guided Nerve-Preserving Surgery
Source: Biomolecules. 2023 Jun 5;13(6):942. doi: 10.3390/biom13060942 (PMC10296720; doi:10.3390/biom13060942)
Supplement: Supplementary file 1 [file biomolecules-13-00942-s001.zip › biomolecules-2350022-supplementary.pdf]

biomolecules

Results

Mass spectometry

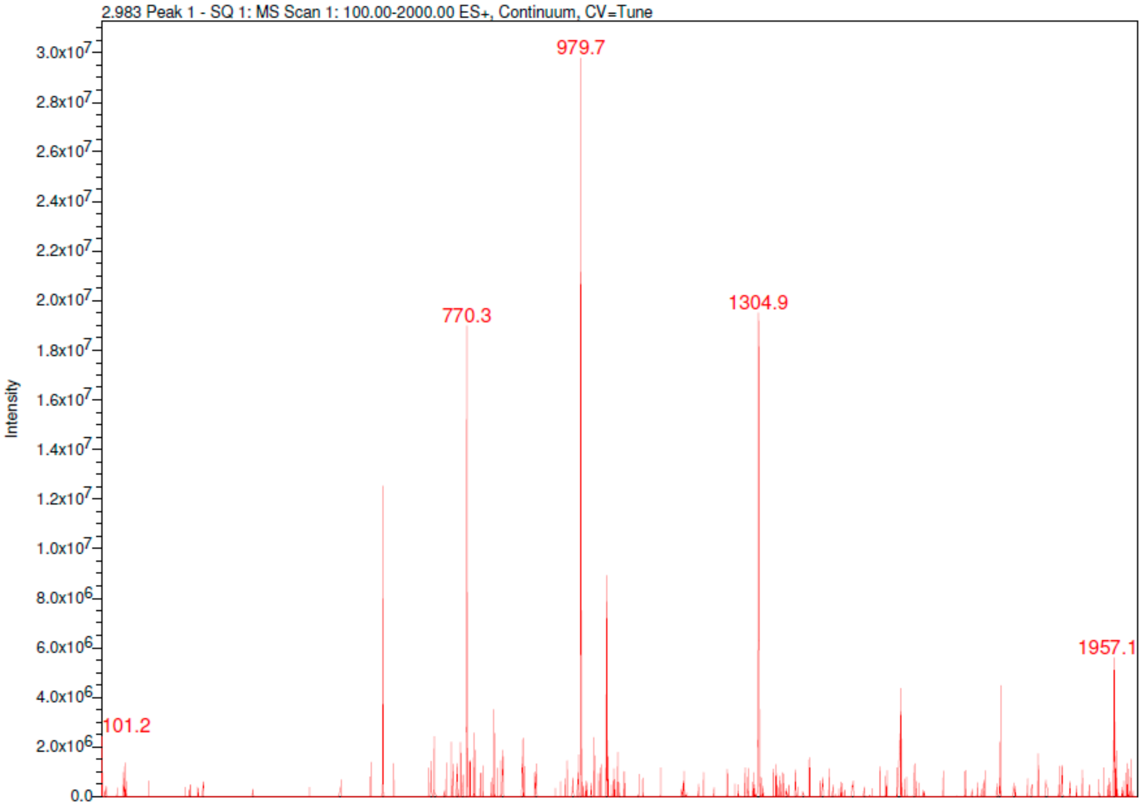

Figure S1. Mass spectrum of Cy5-C-P0$_{101-125}$. m/z [M+3H – 979.7].

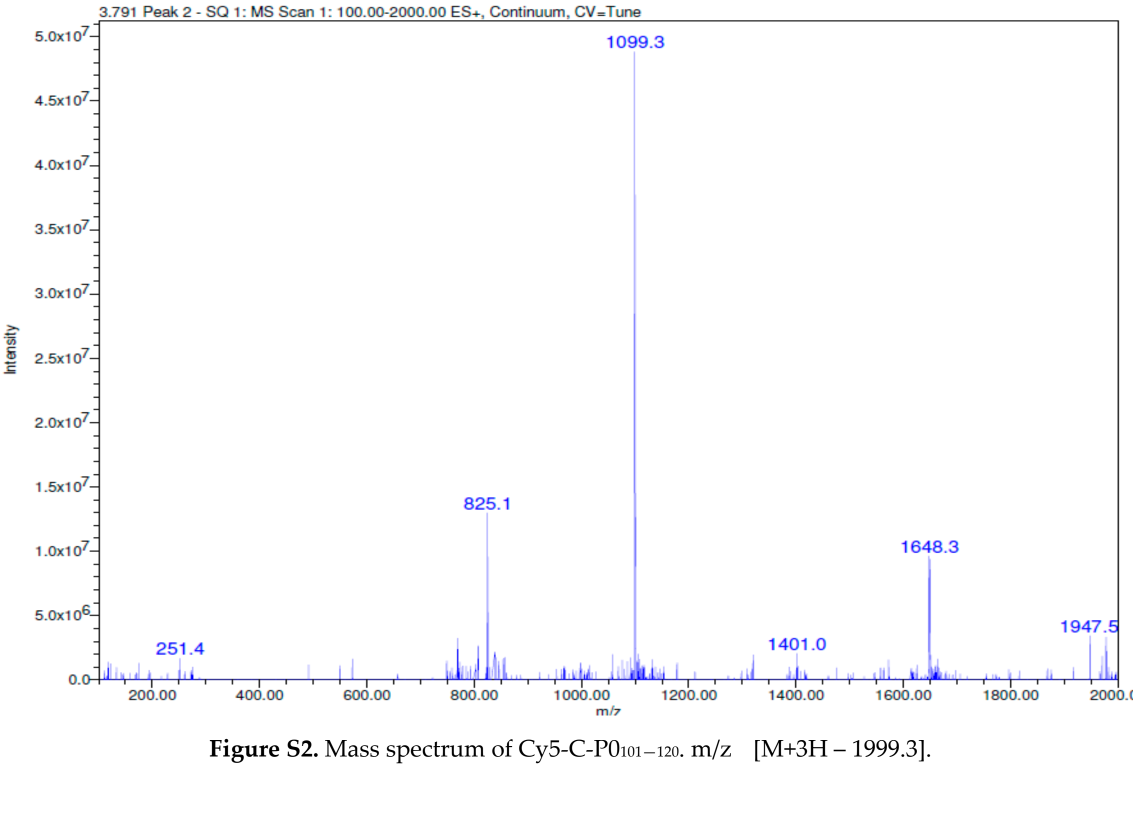

Figure S2. Mass spectrum of Cy5-C-P0$_{101-120}$. m/z [M+3H – 1999.3].

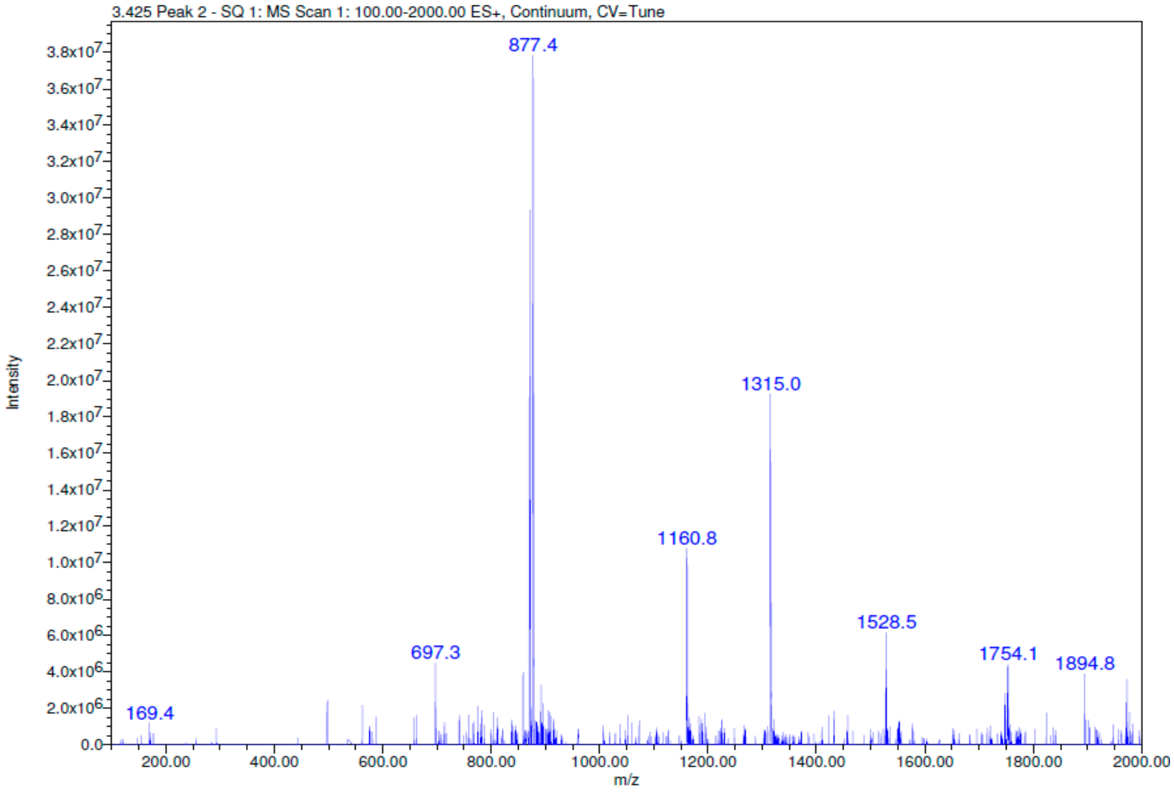

Figure S3. Mass spectrum of Cy5-C-P0$_{101-115}$. m/z [M+3H – 877.4].

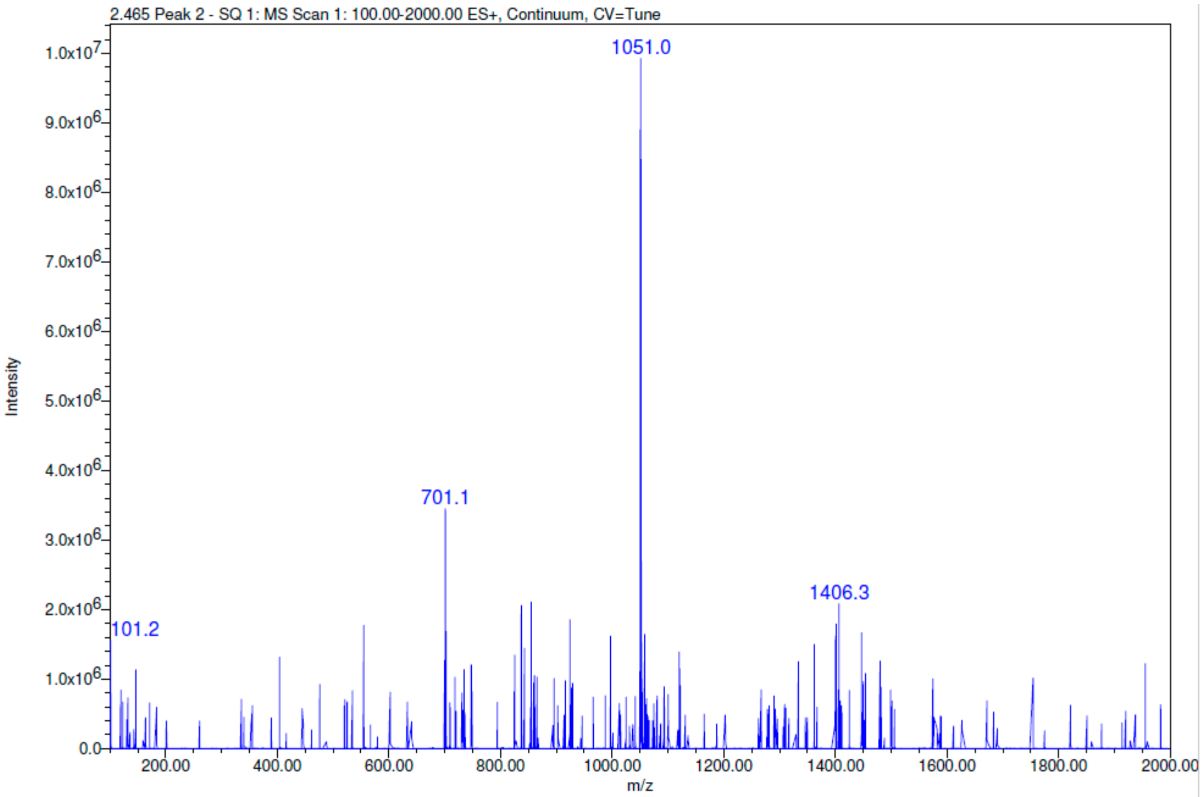

Figure S4. Mass spectrum of Cy5-C-P0$_{101-110.}$ m/z [M+2H –1051.0].

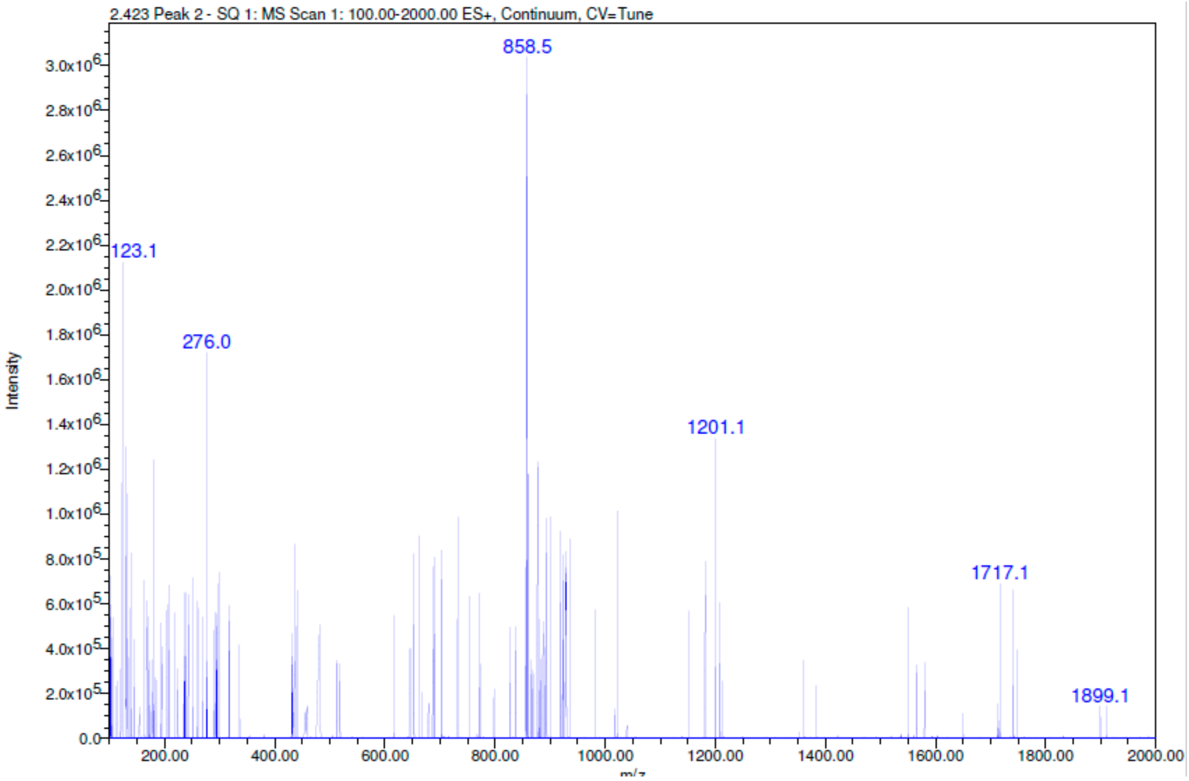

Figure S5. Mass spectrum of Cy5-C-P0$_{101-106}$. m/z [M+2H – 858.5].

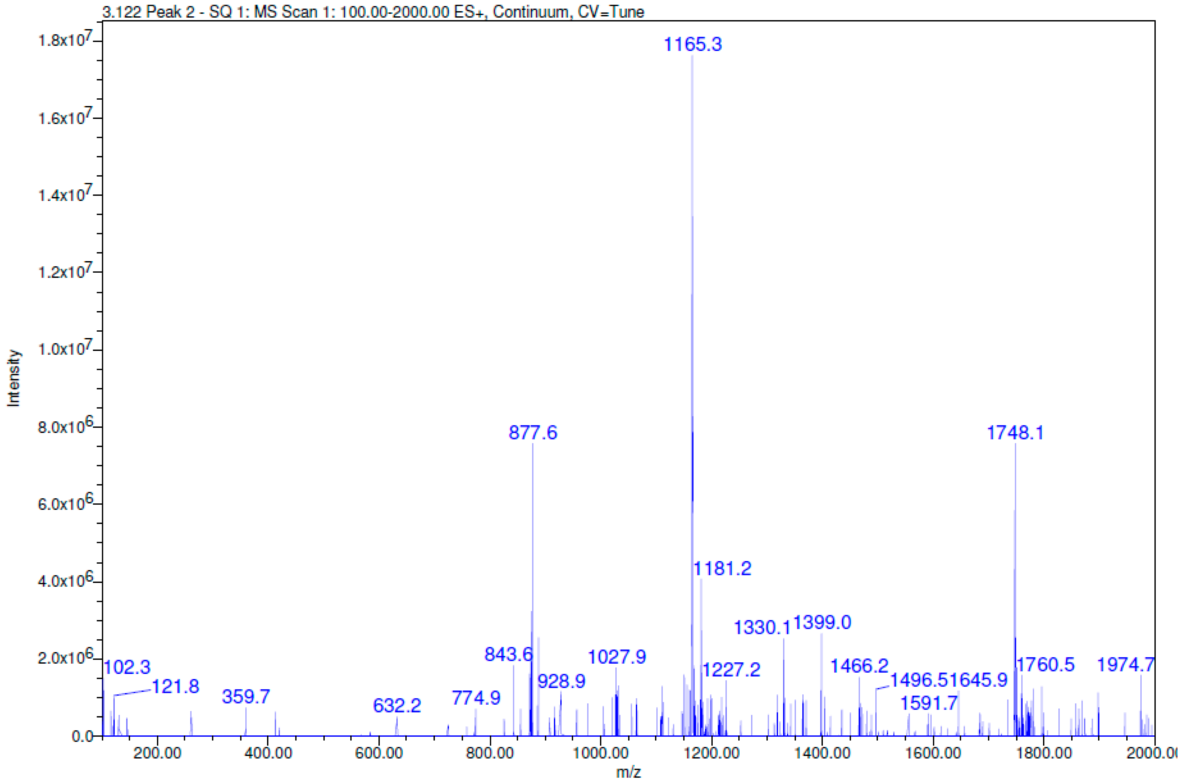

Figure S6. Mass spectrum of Cy5-C-P0$_{105-125}$. m/z [M+3H+ Na$^+$ – 1165.3].

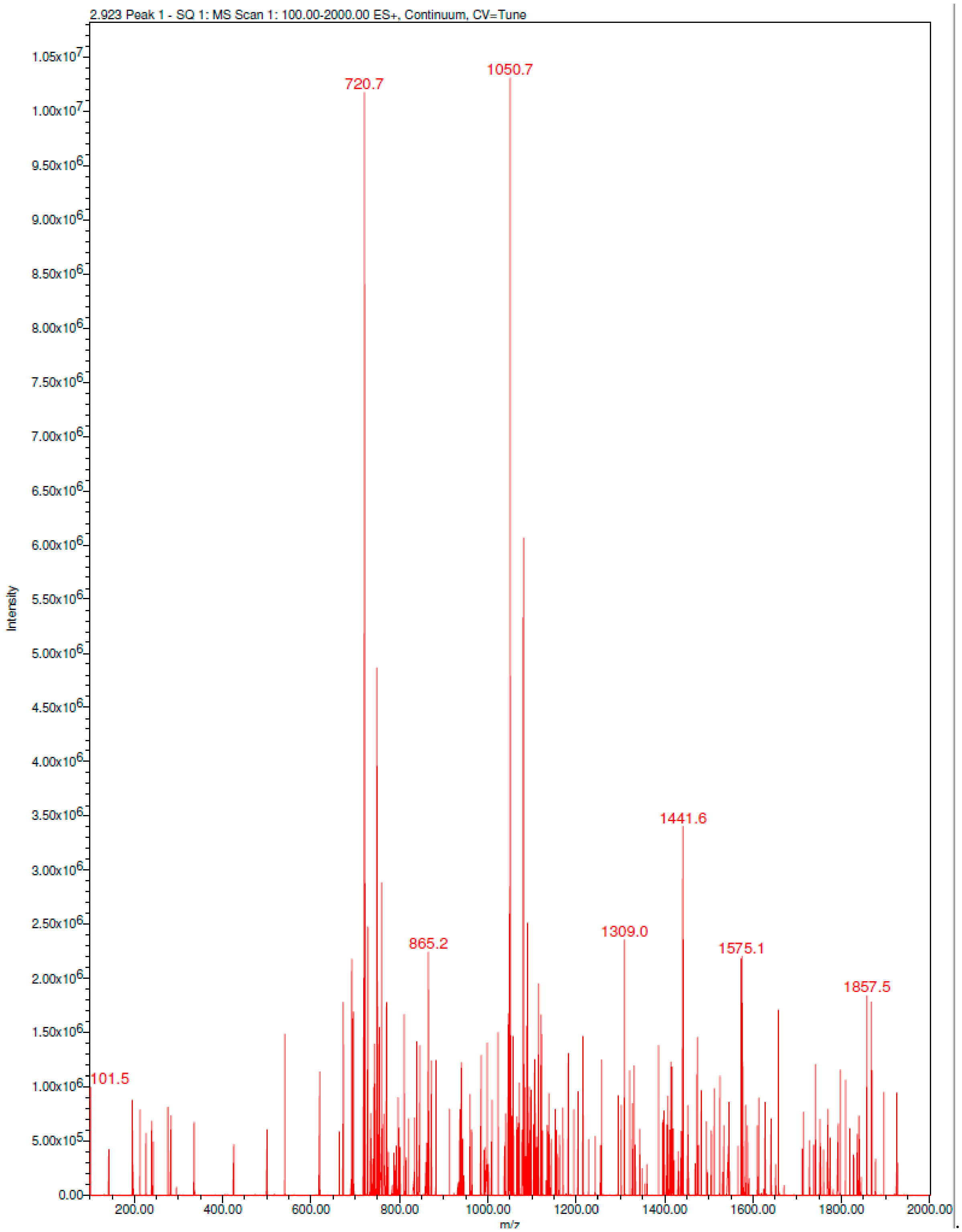

Figure S7. Mass spectrum of Cy5-C-P0$_{108-125}$. m/z [M+2H– 1050.7].

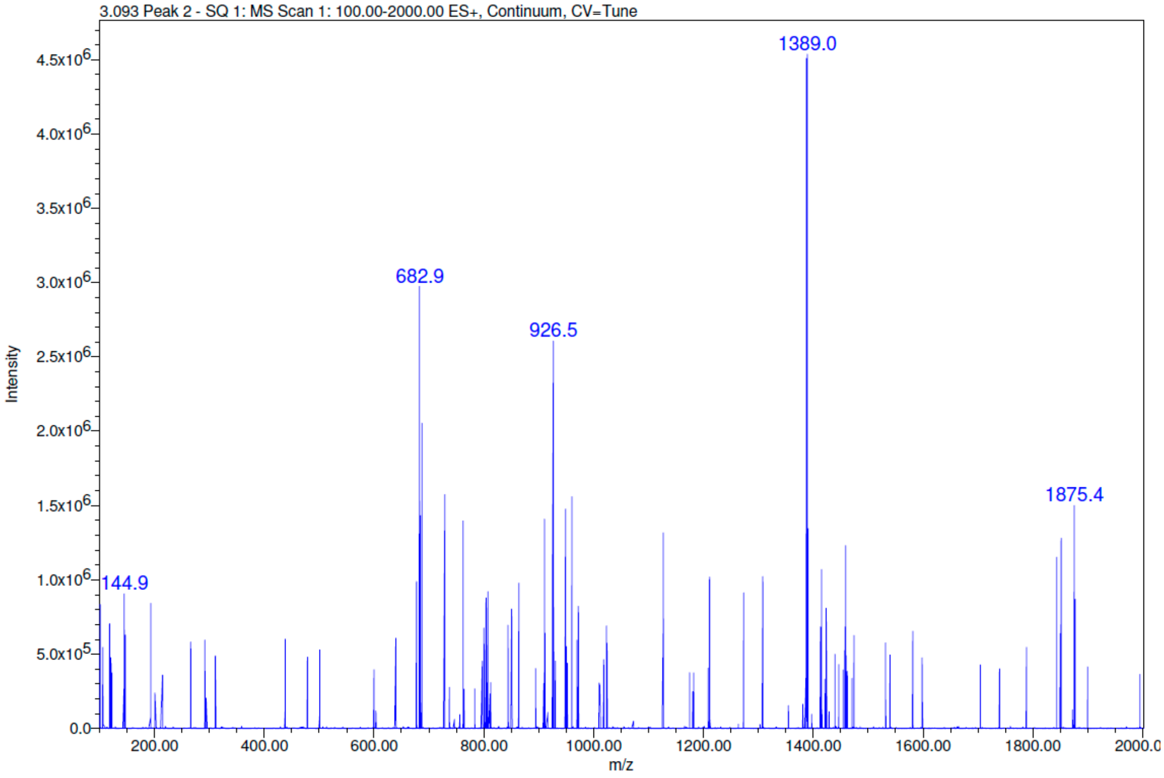

Figure S8. Mass spectrum of Cy5-C-P0$_{112-125}$ .m/z [M+2H – 1389.0].

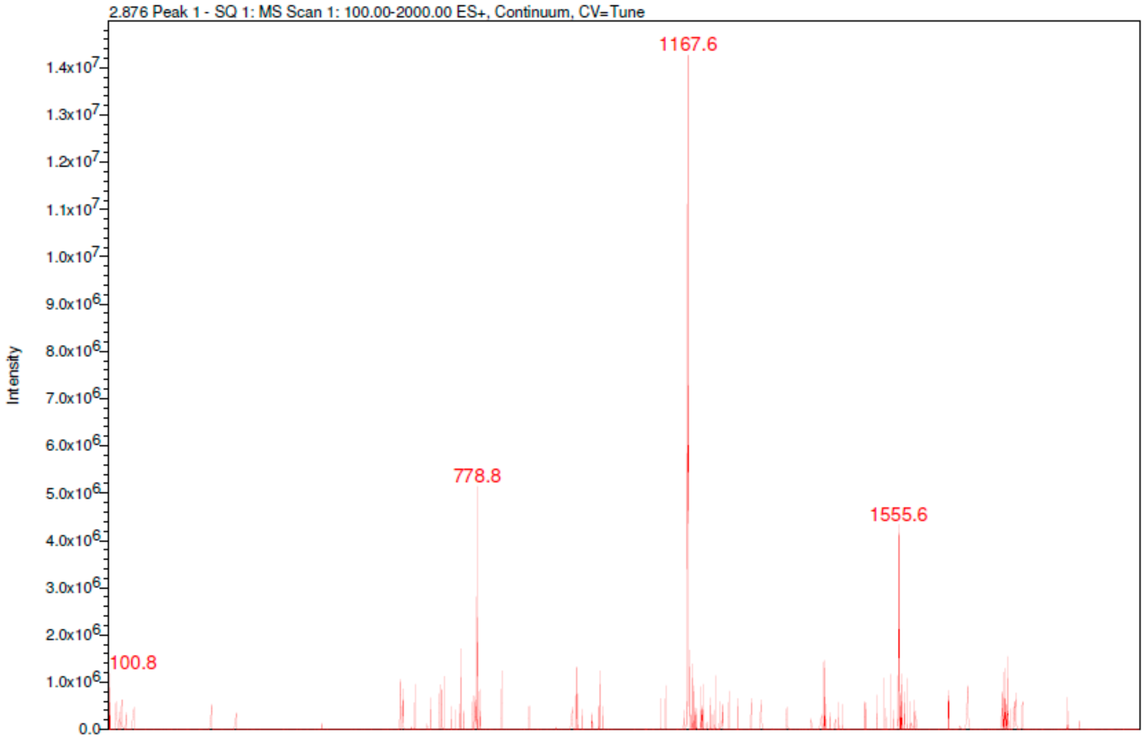

Figure S9. Mass spectrum of Cy5-C-P0$_{116-125}$. m/z [M+2H – 1167.6].

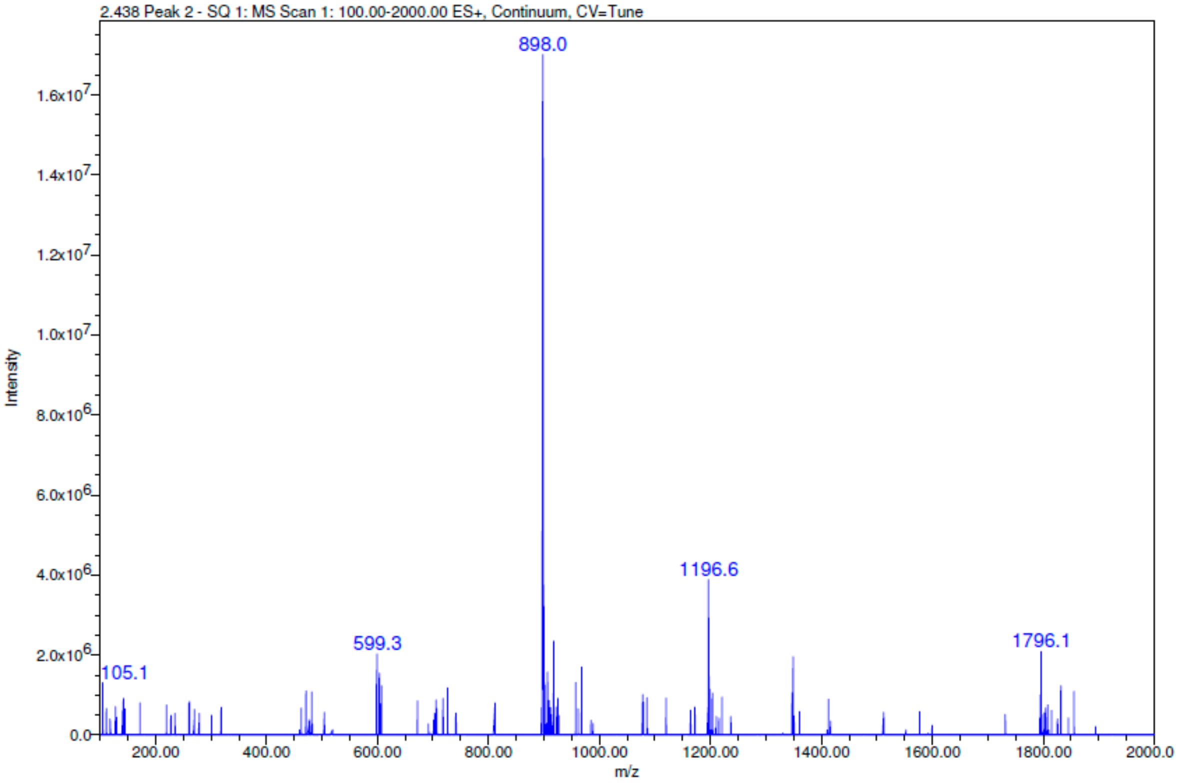

Figure S10. Mass spectrum of Cy5-C-P0$_{120-125}$. m/z [M+2H – 898.0].

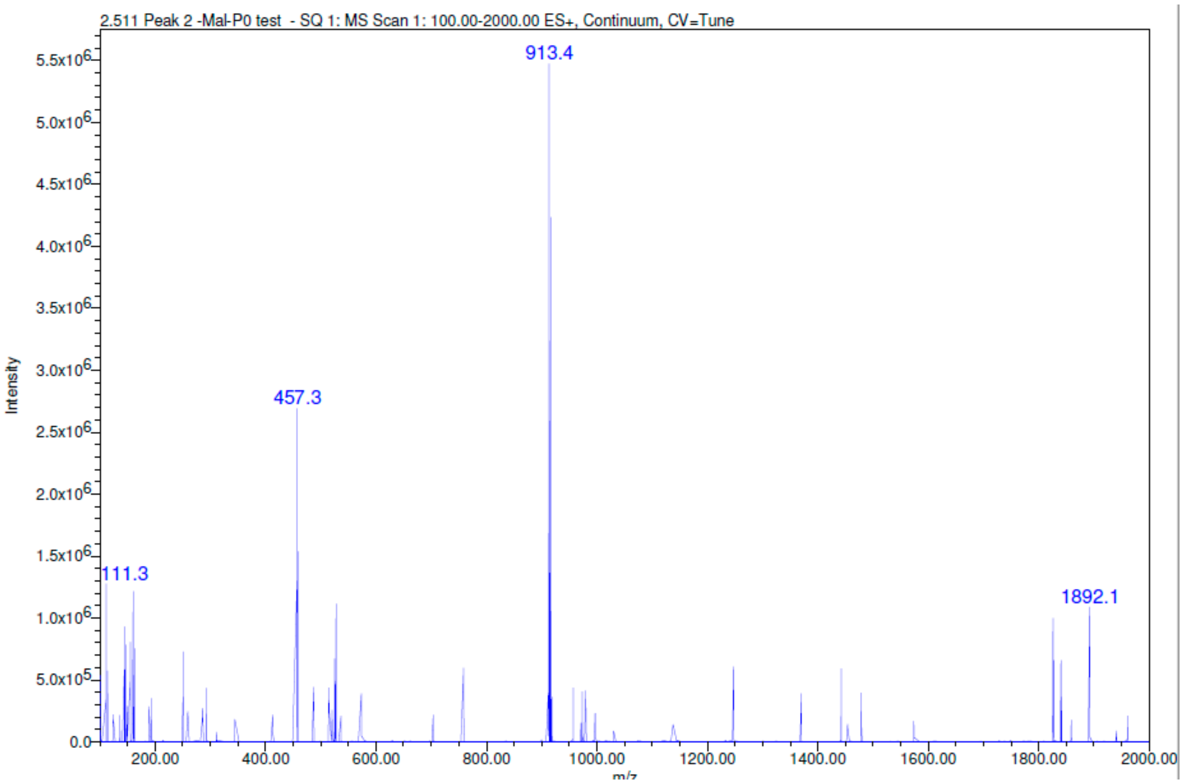

Figure S11. Mass spectrum of *Maleimide-(SO$_3$)Cy5(SO$_3$)Sulfonate*. m/z [M – 913.4].

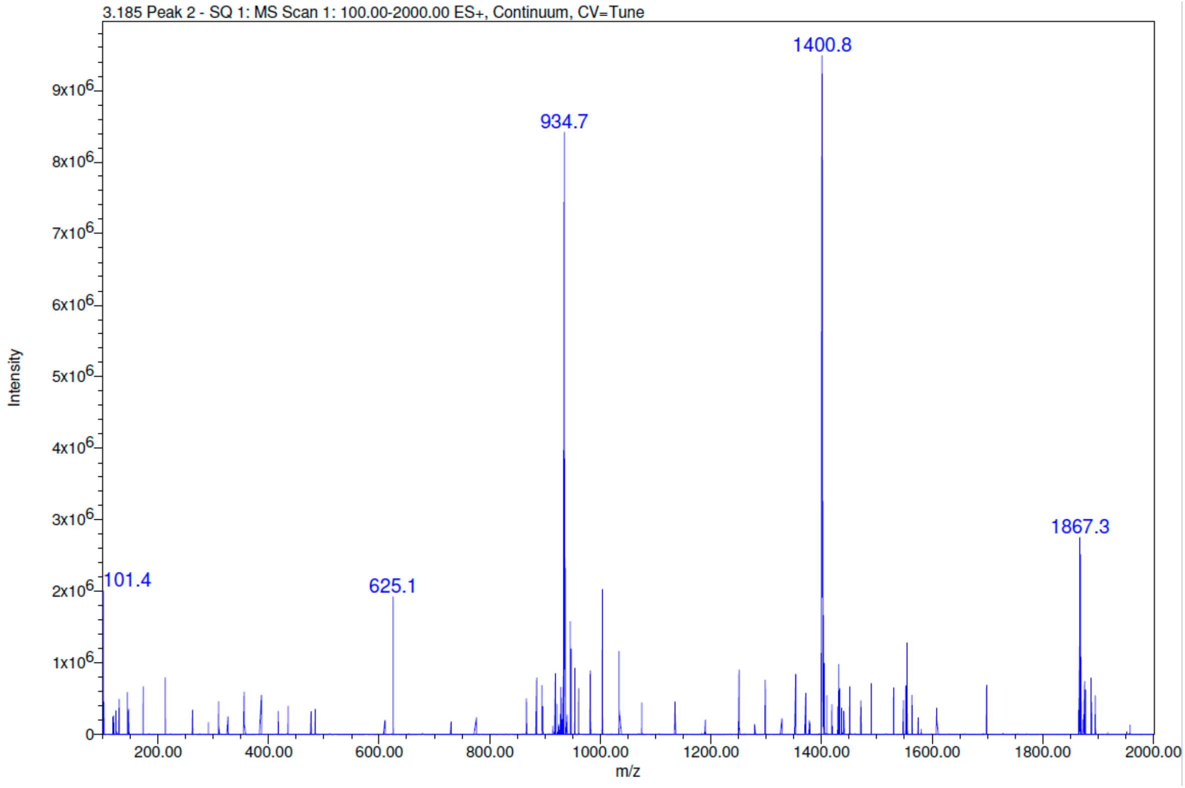

Figure S12. Mass spectrum of Cy7-C-$P0_{112-125}$. m/z [M+2H – 1400.8].

Co-localization P0 staining and lysosomal and nuclear control staining

(3D) surface plot analysis was used to evaluate the localization of the P0-specicic staining further (Figure S13). For peptides with a nanomolar affinity (Cy5-$P0_{101-125}$, Cy5-$P0_{105-125}$, Cy5-$P0_{112-125}$ and Cy5-$P0_{112-125}$) distribution over the cellular outgrowths was confirmed, with limited overlay with the intracellular lysosomal control staining (overlay Cy5/lysosomes in yellow). No significant co-localization with the nulear control staining (in blue) was seen. Moreover, increasing affinity (in particular for Cy5-$P0_{112-125}$ and Cy5-$P0_{112-125}$) resulted in increased fluorescence peak intensities within the Cy5-related staining pattern.

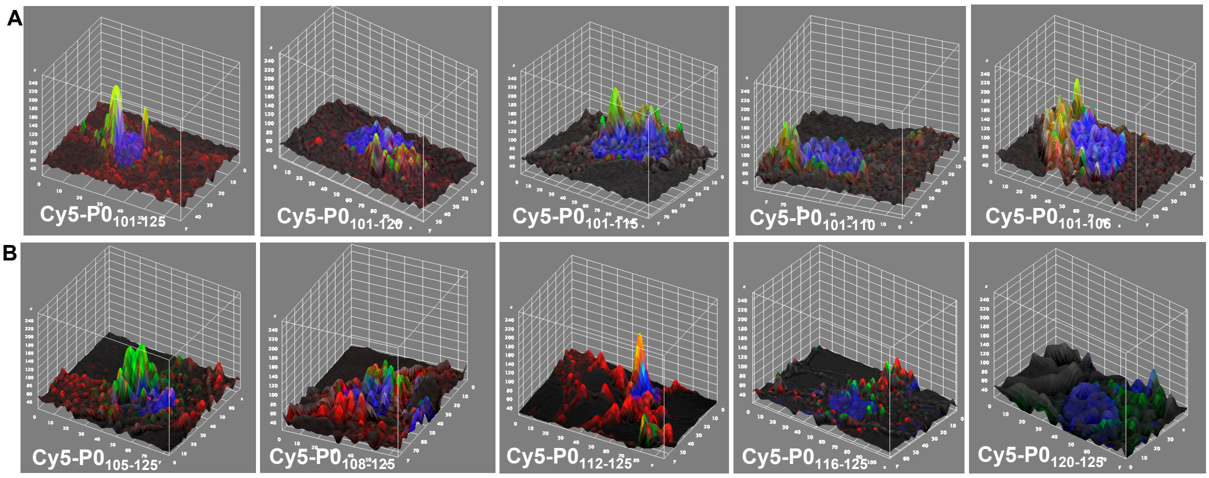

Figure S13. Surface plot analysis fluorescence confocal images Surface plot analysis of the staining pattern throughout the cells for A) the C-terminal matrix and B) the N-terminal matrix. Peptide-related staining (Cy5) in red, lysosomes in green and nuclear staining in blue. Yellow line = orientation 3D analysis.

Semi-quenatitative assessment of the level of co-staining within the fluorescence confocal images (Table S1) revealed an increasing trend of co-localization of lysosomal intracellular staining with decreasing affinity (Figure 3). Co-localization with the nuclear control staining was comparably low for all compounds.

Semi-quantitative assessment of the co-localization of the P0-related staining of Cy5-$P0_{101-125}$ and the intracellular lysosomal (green) was shown to be low for this high affinity peptide (co-localization factor: 0.29; Table S1), while for Cy5-$P0_{101-120}$, Cy5-$P0_{101-115}$, Cy5-$P0_{101-110}$ and Cy5-$P0_{101-106}$ co-localization of signal was more prominent (co-localization factor of respectively 0.42, 0.59, 0.78 and 0.77). Co-localization with the nuclear (blue) control staining was low for all compounds. The absence of staining of outgrowths combined with the higher level of lysosomal co-localization would therefore suggest that the relatively low Cy5-related fluorecsence signal obtrained after C-terminal truncation can be contributed to non-specific staining.

Similar to the results obtained C-terminal matrix, the level of staining of the outgrowths was shown to correspond to the level of co-localization with the intracellular control staining/non-specific staining (Table S1). The lysosomal co-localization factor for Cy5-$P0_{105-125}$ (0.32), Cy5-$P0_{108-125}$ (0.27) and Cy5-$P0_{112-125}$ (0.21) was comparable or lower than the original lead compound, whereas a loss in P0-related staining resulted in increased co-localization for Cy5-$P0_{116-125}$ (0.40) and Cy5-$P0_{120-125}$ (0.39).

Table S1. Semi-quatitative assessment of co-localization staining P0 tracers and lysosomal and nuclear control staining.

	**Co-localization factor (scale: 0-1)**	
**Peptide**	**Cy5 vs Lysosomes**	**Cy5 vs nucleus**
Cy5-$P0_{101-125}$	0.29 ± 0.03	0.14 ± 0.03
Cy5-$P0_{101-120}$	0.42 ± 0.01	0.06 ± 0.02
Cy5-$P0_{101-115}$	0.59 ± 0.20	0.78 ± 0.13
Cy5-$P0_{101-110}$	0.77 ± 0.15	0.15 ± 0.06
Cy5-$P0_{101-106}$	0.77 ± 0.12	0.15 ± 0.06
Cy5-$P0_{105-125}$	0.32 ± 0.0.4	0.07 ± 0.04
Cy5-$P0_{108-125}$	0.27 ± 0.11	0.05 ± 0.04
Cy5-$P0_{112-125}$	0.21 ± 0.06	0.11 ± 0.01
Cy5-$P0_{116-125}$	0.40 ± 0.01	0.04 ± 0.02
Cy5-$P0_{120-125}$	0.39 ± 0.06	0.07 ± 0.02
